# Clinical Ophthalmology Consultations in Paediatric Inpatient Rehabilitation—A Retrospective Case‐Note Analysis

**DOI:** 10.1155/joph/8341531

**Published:** 2026-06-29

**Authors:** Milena Geissbühler, Brigitte Simonsz-Toth, Andreas Meyer-Heim, Christina Gerth-Kahlert

**Affiliations:** ^1^ Medical School, University of Zurich, Zurich, Switzerland, uzh.ch; ^2^ Department of Ophthalmology, University Hospital Zurich, Zurich, Switzerland, usz.ch; ^3^ Swiss Children’s Rehab, Affoltern Am Albis, Zurich, Switzerland; ^4^ University Children’s Hospital Zurich, Zurich, Switzerland, swisshealth.ch; ^5^ University of Zurich, Zurich, Switzerland, uzh.ch; ^6^ Children’s Research Centre, University Children’s Hospital Zurich, Zurich, Switzerland, swisshealth.ch; ^7^ Department of Ophthalmology, University Hospital and University of Zurich, Zurich, Switzerland, usz.ch

**Keywords:** cerebral visual impairment, children, paediatric, rehabilitation

## Abstract

**Background:**

One of the key factors for enhancing outcomes in neurorehabilitation of patients with congenital or acquired brain injury is optimal sensory function and identification of possible visual dysfunctions. Awareness of visual dysfunctions including cerebral visual impairment (CVI) is therefore of utmost importance in the rehabilitation process. The purpose was to investigate the frequency and nature of vision disorders in children admitted to a rehabilitation centre over a 5‐year period.

**Methods:**

This is a retrospective analysis of patients at the Swiss Children’s Rehab who received an ophthalmological consultation between January 2019 and December 2024. Electronic chart review was performed in patients with consent for the study.

**Analysed Data:**

Referral diagnoses leading to admission to the rehabilitation centre, corresponding ophthalmological findings, therapeutic interventions and demographics. The study was approved by the local ethics committee.

**Results:**

A total of 229 consultations from 163 patients were reviewed. The two most common causes for admission were traumatic (41/163) and inflammatory (36/163) conditions. Congenital disease was present in 30 patients. Most of the children had an acquired disorder (133/163). Pathological ophthalmological findings were present in 101/163 (62%) children examined. The most common findings were reduced vision (26/163) followed by CVI (22/163) and refractive errors (20/163). Traumatic causes were most often associated with oculomotor disorders (10/41) or CVI (6/41). CVI was prevalent in 22% of the pathological findings in this study.

**Conclusions:**

Ophthalmological assessment is recommended for all children with suspected visual dysfunction as part of the rehabilitation process.

## 1. Introduction

Recovery in patients with trauma, severe congenital or acquired disease or malformation requires optimal sensory function and identification of possible vision deficits. The capacity for optimal visual function is a fundamental aspect of children’s developmental progress, influencing not only social and emotional abilities [1] but also motor development [2]. In general, early diagnosis and treatment of the visual dysfunction and appropriate adaptation of the environment will lead to a significant improvement in visual function [3].

Damage to the brain is one of the most common causes of cerebral visual impairment (CVI) and has been identified as the predominant cause of low vision in children within Western countries [4]. The definition of cortical and CVI is used interchangeably: While it may be distinguished between cortical and subcortical vision impairment with the characteristic neuro‐ophthalmological signs defined by Brodsky et al. [5], others argue that cortical injuries do not occur without subcortical changes [6]. The aetiology of CVI can vary significantly. The most prevalent cause is hypoxic–ischaemic injuries; although, other potential aetiologies include brain trauma [7], epilepsy, malformations, infections of the central nervous system, metabolic diseases, poison or drugs and neurodegenerative diseases [8, 9]. CVI has been defined as ‘a verifiable visual dysfunction which cannot be attributed to disorders of the anterior visual pathways or any potentially co‐occurring ocular impairment’ [10]. This definition was confirmed in the European consensus statement by Lindquist and Westerberg [11]. In contrast to visual impairments caused by ocular diseases, CVI does not represent a clearly defined single condition but rather encompasses a range of distinct visual processing disorders [6]. An important differential diagnosis of CVI is delayed visual maturation (DVM), which is typically diagnosed retrospectively and is characterised by improvement in visual function over time [12], mainly through neural plasticity during the neonatal period.

Thus, awareness and adequate assessment of CVI and other visual dysfunctions are key elements in the rehabilitation process. From a clinical ophthalmology perspective, understanding the frequency, indications and spectrum of ophthalmological findings in this setting is essential to support diagnosis and interdisciplinary management. However, the prevalence of CVI and visual disorders in children attending an inpatient rehabilitation clinic has not been assessed. The purpose of this study is to analyse the visual function and corresponding ophthalmological findings according to the admission diagnosis in patients at the Swiss Children’s Rehab over a 5‐year period with consideration of CVI as part of the overall spectrum of the ophthalmological findings. To delineate these data, results were grouped according to the reasons for, and diagnoses leading to, admission to inpatient rehabilitation.

## 2. Methods

This is a retrospective analysis of patients admitted to the Swiss Children’s Rehab who received an ophthalmological consultation between 01 January 2019 and 31 December 2024. The dataset included children and adolescents aged 0–20 years, who were hospitalised at the Swiss Children’s Rehab between 01 January 2019 and 31 December 2024 and received a consultation from the team of the Department of Ophthalmology at the University Hospital Zurich. The Swiss Children’s Rehab is a 47‐bed inpatient facility for paediatric rehabilitation. It is a comprehensive paediatric rehabilitation centre, with neurorehabilitation representing one of its specialised services. Children are referred from various centres across Switzerland. The Rehab centre provides inpatient rehabilitation for children with both congenital and acquired diseases and injuries.

### 2.1. Participants

The patients examined by the ophthalmological team were triaged by the paediatrician taking care of them at the Rehab centre. Indications were (1) known preexisting ocular diseases, (2) diseases or trauma that had directly involved the eye and or the visual pathway, (3) new visual deficits observed by the caretaker, clinical and rehabilitation staff or teachers at the Rehab centre, (4) unknown visual function due to reduced general health status, and (5) visual deficits noticed during a low vision assessment performed as a standard. The paediatric team informed the ophthalmological team about the general diagnosis and purpose of the assessment. The age of the patient was not relevant for referral. The time of the consultation in respect to the injury date or illness onset was chosen depending on general health status, visual function, and the question to be answered. For example, a child with severe head trauma was seen very soon after the accident to rule out visual loss. A child after an acute infection was seen later once the therapist realised a deficit in reading. The orthoptist and ophthalmologist decided which examinations to perform depending on the general health status and cooperation of the child. Follow‐up appointments were scheduled depending on the diagnosis. They were automatically scheduled by the ophthalmological team for one of the next clinical visits. Once the child was discharged from the Rehab centre, follow‐up appointments were organised close to their hometown.

### 2.2. Measures

The data analysed included the following information:

1. Referral diagnoses leading to admission to the inpatient rehabilitation.
2. Ophthalmological findings established during the ophthalmological assessment. The ophthalmological team did not have an examination suite available at the Swiss Children’s Rehab. The team of ophthalmologists and orthoptists used portable examination material and instruments at each visit. Visual acuity tests were chosen depending on age, general developmental status, rehabilitation progress and main diagnostic question. Often, children showed a low attention and concentration phase. Thus, not all tests were performed. Cardiff charts, the Lea crowding 25% test or the Landolt C test (crowding optotype presentation) were used for near vision assessment, whereas the Lea test or Landolt C test were used for distance vision assessment. Orthoptic examination included tests of ocular motility, stereoacuity (using Lang 1, Lang 2 tests), cover tests at distance and near and the red reflex test. In addition, pupillary response to light, convergence, and confrontational visual fields were assessed. Ophthalmological checkup included the anterior segment and undilated or dilated fundus examination using a hand‐held slit‐lamp and indirect ophthalmoscopy with a 20D or 28D lens, retrospectively. Intraocular pressure was measured using a hand‐held device. Refraction was measured by retinoscopy or automatically by a refractometer after instilling cycloplegic eye drops (tropicamide or cyclopentolate depending on the contraindications of each child).
3. Ophthalmological therapeutic interventions including glasses, amblyopia therapy, topical medication (eye drops/ointment), watch‐glass bandage, initiation of low vision support and recommendation for training for visual field defect compensations. Therapeutic recommendations documented in the medical reports by the responsible ophthalmologist were systematically extracted to assess the proportion of children in whom a therapeutic measure could be implemented during the inpatient rehabilitation period.

Demographic variables were also recorded (age at examination and sex). The population was divided into equally sized 3‐year age intervals to allow better interpretation and comparison of absolute case numbers across referral diagnoses. For the analysis of ophthalmological findings, age groups were defined according to specific steps in visual maturation: 0–2, 3–6, 7–10 and 11–20 years, reflecting clinically relevant phases of visual development.

Multiple consultations in one patient were assessed independently but excluded from the cumulative case count. The referral diagnoses were categorised as either congenital or acquired (Table 1). Vascular malformations, including cavernous malformations and aneurysms associated with acute haemorrhagic stroke, were classified as congenital. The referral diagnoses were further classified into the following categories: neoplasm, epilepsy, vascular, malformation, inflammatory, trauma and others (Table 1). The ‘trauma’ category included cases of isolated traumatic brain injury (TBI), polytrauma, and trauma‐related hypoxic–ischaemic encephalopathy. In the group of acquired referral diagnoses, the category ‘others’ included children with a diagnosis not clearly assignable to any of the predefined categories. This group comprised patients who underwent rehabilitation following non‐neurosurgical procedures and cases of encephalopathy without a clearly identifiable cause. The congenital ‘others’ category encompassed preterm infants, children with metabolic or syndromic disorders, postoperative courses, and patients with an unclear diagnosis. Ophthalmological findings comprised both structural ocular diagnoses and functional visual impairments. For the purposes of this study, ‘visual dysfunction’ was defined as a collective term encompassing all clinically relevant abnormalities affecting visual function. The findings were classified into the following categories, whereas more than one category could be assigned to each patient: normal, CVI, amblyopia, oculomotor disorders, opticopathy, refractive errors, reduced vision (not related to CVI), strabismus, keratopathy, visual field loss, and examination not possible (Table 1). The group normal includes patients where no abnormalities were detected. The category reduced vision refers specifically to reduced visual acuity as assessed in the context of age. Therapeutic recommendations documented in the medical reports by the responsible ophthalmologist were systematically extracted, as shown in Table 1, to evaluate the proportion of children in whom a therapeutic intervention was initiated during inpatient rehabilitation.

**TABLE 1:** Overview of analysed data.

Referral diagnoses	Ophthalmological findings	Therapeutic interventions
• Congenital	• Normal	• Occlusion therapy (initiated)
• Acquired	• Amblyopia	• Occlusion therapy (continued)
Each further subclassified as follows:	• Oculomotor disorders	• Occlusion therapy (stopped)
• Neoplasm	• Opticopathy	• New or adjusted spectacle prescription
• Epilepsy	• Strabismus	• Lubricating eye drops/watch‐glass bandage
• Vascular	• Keratopathy	• Initiation of low‐vision support
• Malformation	• Visual field loss	• Training for visual field defects
• Inflammatory	• Refractive errors	
• Trauma	• Reduced vision (not related to CVI)	
• Others	• CVI	
	• Examination not possible	

*Note:* This table presents the classification of referral diagnoses, ophthalmological findings and therapeutic interventions.

Abbreviation: CVI = cerebral visual impairment.

The diagnosis of CVI is based on a comprehensive clinical assessment and should be assessed and established by a multidisciplinary team, with ophthalmological examination forming an essential component [11]. Clinical assessment of CVI typically includes evaluation of relevant neurological risk factors, evaluation of visual behaviour and evidence of visual dysfunction [13]. Risk factors may include birth injuries, infections or structural malformations [13]. In the present study, this aspect was analysed based on the referral diagnosis. Assessment of visual behaviour may reveal abnormalities in various domains, including difficulties locating objects, clumsiness or difficulties recognising people, shapes or places, as well as difficulties processing moving visual stimuli [13]. Evidence of visual dysfunction may include findings such as an abnormal crowding test, ocular motor abnormalities or visual field defects [13]. In this study, visual dysfunction was evaluated based on documented ophthalmological findings without additional neuropsychological assessment.

### 2.3. Data Collection and Analysis

An electronic chart review was performed on patients with available consent for the study. The medical reports, which were documented by the ophthalmological team of the University Hospital Zurich, were systematically reviewed, and predefined variables were extracted for analysis. After the initial entry of data into the data collection file by M.G., a reanalysis was conducted for quality assurance purposes to ensure data accuracy by Milena Geissbühler and Christina Gerth‐Kahlert. Subgroup categories for referral diagnoses, ophthalmological findings and demographic variables were predefined prior to the chart review. In contrast, the categorisation and analysis of therapeutic interventions were developed post hoc after review of the documented measures, based on the therapeutic actions recorded in the medical reports. The study was descriptive in nature. Categories were analysed using case numbers and frequency distributions. All data were entered into an Excel file and analysed across different dimensions of the predefined study categories. ChatGPT (OpenAI GPT‐5) was used to assist in generating *R* code for the analysis of the data presented.

### 2.4. Ethics Approval

The study was authorised by the Cantonal Ethics Committee Zurich (BASEC‐Nr. 2024‐01779).

### 2.5. Consent to Participate

Only patient data were included if either written general consent of the University Children’s Hospital/Swiss Children’s Rehab was available or the general consent was unavailable or declined, but specific written study consent for the use of ophthalmological data in this study was agreed to by the parents or legal guardians. Children for whom general consent was not available and whose parents declined specific consent for the use of ophthalmological data were not included in the study analysis. Furthermore, in cases where general consent was missing and the child had passed away, no contact was initiated with the parents, and these patients were not included.

## 3. Results

**FIGURE 1:**
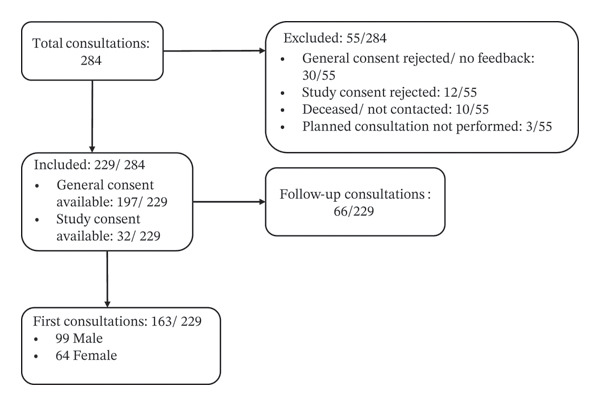
Flowchart of consultation analysis. This flowchart presents an overview of all consultations and identifies those included in the study or analysed separately as follow‐up consultations during the analysed time period.

A total of 284 ophthalmological consultations were scheduled at the Swiss Children’s Rehab during the study period. Of these, 229 of 284 consultations were included in the data analysis. The remaining consultations were excluded because of missing consent (52/284) or missed appointments (3/284). Of the analysed consultations, 66 of 229 were follow‐up appointments as illustrated in Figure 1. The proportion of female patients was 39% (64 female and 99 male), as presented in Table 2.

**TABLE 2:** Demographic characteristics and age distribution of the study population.

Variable	Value
Age at examination, years, median (IQR)	9.41 (4.96–12.9)

*Age group, years, n (%)*	
0–2 years	24 (14.7%)
3–5 years	25 (15.3%)
6–8 years	32 (19.6%)
9–11 years	26 (16.0%)
12–14 years	36 (22.1%)
15–17 years	18 (11.0%)
18–20 years	2 (1.2%)

*Sex, n (%)*	
Female	64 (39.3%)
Male	99 (60.7%)

*Note: n*, number of patients.

Acquired conditions were the most common cause for admission to the rehabilitation centre (133/163) including trauma history (31%) followed by inflammatory diagnosis (26%) as summarised in Table 3. Among the congenital cases, the underlying referral diagnosis was predominantly the vascular category (37%).

**TABLE 3:** Overview of referral diagnoses.

Referral diagnosis	All (*n*)	Congenital category (*n*)	Acquired category (*n*)
Total	163	30	133
Neoplasm	14	0	14
Epilepsy	19	3	16
Vascular	22	11	11
Malformation	7	7	0
Inflammatory	36	1	35
Trauma	41	0	41
Others∗	24	8	16

*Note: n*, number of patients.

^∗^Includes postoperative cases (non‐neurosurgical), preterm births, metabolic disorders, syndromic conditions, encephalopathy and unclear diagnoses.

A pathological ophthalmological diagnosis was identified in 62% of the children, with multiple diagnoses possible in some of them. The three main diagnoses were reduced vision (26/163), followed by CVI (22/163) and refractive errors (20/163). The frequency distribution of ophthalmological findings varied according to the underlying referral diagnosis as listed in Table 4. Reduced vision not related to CVI was the most prevalent ophthalmological finding in both categories of acquired and congenital conditions. CVI was the second and third most observed ophthalmological finding in those categories, respectively. Oculomotor disorders ranked third among the acquired diagnosis. Refractive errors were the second most common diagnosis in the congenital referral group.

**TABLE 4:** Overview of ophthalmological findings.

Finding	All /(*n*)	Congenital category (*n*)	Acquired category (*n*)
Total findings	204	40	164
Normal	62	12	50
CVI	22	5	17
Amblyopia	7	1	6
Oculomotor disorders	16	0	16
Opticopathy	17	3	14
Refractive	20	6	14
Reduced vision	26	7	19
Strabismus	11	2	9
Keratopathy	8	2	6
Visual field loss	11	2	9
Examination not possible	4	0	4

*Note: n*, number of documented diagnoses.

Abbreviation: CVI = cerebral visual impairment.

Subgroup analysis revealed oculomotor dysfunction (10/41) as the most frequent ophthalmological finding in patients with the referral diagnosis ‘trauma’, followed by CVI (6/41). In contrast, for inflammatory referral diagnoses, the most common ophthalmological findings were CVI (7/36) and reduced vision (7/36).

**FIGURE 2:**
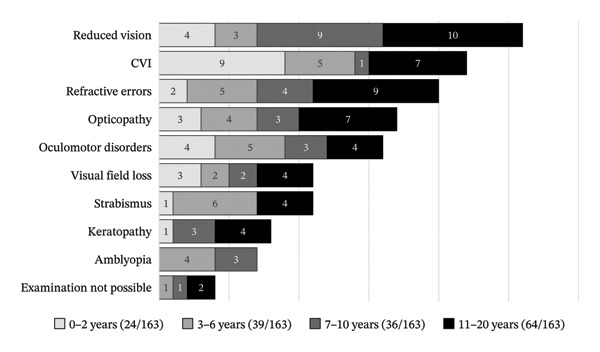
Distribution of ophthalmological findings according to age. This diagram illustrates the frequency of various ophthalmological findings among patients, divided into four age groups according to specific steps in visual maturation. CVI, cerebral visual impairment.

The analysis of the age distribution using 3‐year intervals revealed the highest number of patients in the age group 12–14 years (36/163) as listed in Table 2. The 18–20‐year age group was the least represented (2/163). Traumatic causes were frequent in all age groups. Most inflammatory cases were observed in children aged 6–11 years. Epilepsy was most frequently present among children aged 3–5 years. In the 12–14‐year age group, a notable number of cases were classified under the ‘other’ category. All these children had been hospitalised for rehabilitation due to postoperative reasons (non‐neurosurgical). Ophthalmological findings were analysed across the four predefined age groups (0–2, 3–6, 7–10, and 11–20 years). The diagnosis of CVI was most frequently made in children aged 0–2 years as depicted in Figure 2.

**FIGURE 3:**
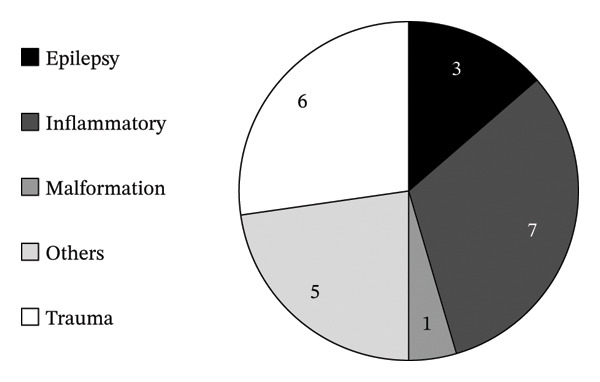
Distribution of disease category in children with cerebral vision impairment. This pie chart illustrates the distribution of referral diagnoses among the 22 patients with cerebral visual impairment.

CVI accounted for 22% of pathological ophthalmological findings. Of these 22 children with CVI, 17 were categorised under an acquired referral diagnosis, while 5 were classified as congenital. The most prevalent underlying cause was inflammatory (7/22), followed by traumatic aetiology (6/22) as illustrated in Figure 3. No child diagnosed with CVI had been referred due to a neoplasm or a vascular condition.

### 3.1. Follow‐Up Consultations

Of the 229 ophthalmological consultations, 66 were follow‐up consultations including 2 (46/66), 3 (16/66), or 4 repeat examinations (4/66). A change was observed in 12 children during follow‐up consultations. An improvement in visual function was documented in 4/12 children, resulting in a normal ophthalmological finding at the follow‐up consultation. In the remaining patients, additional new findings and diagnosis were recorded, including CVI (3/12), visual field defects (2/12), oculomotor disorders (1/12) and refractive errors (1/12). In 2/12 cases, a pathological finding could only be identified during the follow‐up consultation, as a complete examination had not been possible during the initial consultation due to a lack of cooperation.

### 3.2. Therapeutic Recommendations

Following the 229 consultations, various therapeutic interventions were initiated. As a result of the first consultations, occlusion therapy in amblyopia was initiated (12/163), continued (2/163) or stopped (1/163). New or adjusted spectacle prescriptions were recommended for 13/163 patients. Lubricating eye drops or a watch‐glass bandage was prescribed in 11/163 patients. Low vision support was initiated in 12/163 patients. Additional recommendation included training for visual field defect compensation (5/163).

## 4. Discussion

This study provides a first overview of a high prevalence of pathological ophthalmological findings in the inpatient setting of the Swiss Children’s Rehab. In this analysis, 62% of the children examined had pathological ophthalmological findings, with CVI identified in 22% of these cases. Visual dysfunction was particularly common in children with TBI, as well as in those with inflammatory or vascular referral diagnoses. These findings highlight the importance of integrating routine ophthalmological and low vision assessments into standard paediatric rehabilitation protocols.

Overall, reduced vision unrelated to CVI represented the most frequent pathological ophthalmological finding, followed by CVI. In cases with traumatic aetiology, the most observed abnormality was an oculomotor dysfunction. These findings are largely consistent with previous reports. However, any observed differences may reflect the specific clinical characteristics and referral patterns of this specialised inpatient rehabilitation cohort rather than epidemiological variation. Previous studies have shown that oculomotor dysfunction is common after concussion in both adults [14–16] and children and adolescents [14, 17]. In contrast to a previous study that examined the occurrence of oculomotor visual disorders in adults [16], no oculomotor dysfunction was observed in our cohort in association with vascular causes, which may relate to the specific clinical cohort of children in an inpatient rehabilitation centre. In moderate to severe TBI, structural neurological and axonal damage has been increasingly reported, which may lead to oculomotor or optic neuropathies [15]. Mild optic neuropathy may have been underdiagnosed in our cohort due to (1) the lack of long‐term follow‐up and (2) unavailability of retinal nerve fibre layer quantification by optical coherence tomography (OCT) at the rehabilitation centre.

In our cohort, CVI was the second most common ophthalmological finding within the trauma category, which may reflect the underlying structural brain damage. A previous study showed that cerebral visual disorders are frequent after brain injuries (20%–40%) [18]. The most known cause of CVI is hypoxic–ischaemic encephalopathy [19, 20]. In our study, 4 of the 22 children with CVI had hypoxic–ischaemic encephalopathy, mostly of traumatic causes (3/4). Inflammatory causes with secondary brain damage represented the second most common aetiology of CVI within the inpatient rehabilitation cohort. Although infections have rarely been reported as a primary cause in previous studies, certain infectious conditions are recognised as potential contributors to the development of CVI [21–23]. As reported in previous studies, other frequent causes include epileptic seizures after brain injury, prematurity, hydrocephalus, and other structural abnormalities [19, 20]. The fact that CVI was most frequently observed in children aged 2 years and younger in this study is consistent with the most common underlying causes. It should be noted that the diagnosis of CVI at such a young age can only be made with limitations based on the available data. In this study, the classification of CVI was primarily based on the exclusion of peripheral visual impairments. Therefore, in children with abnormal visual responses in the absence of peripheral findings, a central cause was assumed. Given the retrospective design and limited longitudinal data, diagnostic uncertainty in establishing a diagnosis of CVI cannot be entirely excluded. An important differential diagnosis to consider in young children when CVI is suspected is DVM [12]. DVM is characterised by improvement in visual function of different extent, but the diagnosis can only be confirmed retrospectively [12]. Despite the incomplete documentation of medical histories in our study, the diagnosis of CVI in children aged 2 years and younger was only applied when the rehabilitation reports contained no history or indication of abnormal visual development or DVM prior to the incident in the acquired category group. To definitively differentiate CVI from DVM, follow‐up examinations would be required, as DVM, in contrast to CVI, may resolve over time partially or completely, or those children will be diagnosed with CVI later on. However, the lack of follow‐up data from the included children, who were followed after discharge from the Rehab centre in other ophthalmological care centres or in private practice, does not permit to differentiate between children with DVM and spontaneous improvement and those who are classified as affected by CVI.

In this study, only 6% of the follow‐up consultations demonstrated excellent improvement, with full recovery of visual function. However, the timing of follow‐up assessments and their relationship to specific therapeutic interventions were not systematically documented, which limits any conclusions regarding treatment effects. Although previous studies have suggested that targeted therapeutic approaches, including vergence and accommodative training, saccadic and pursuit exercises and, where appropriate, balance and head movement rehabilitation, can substantially enhance visual function after visual impairment resulting from TBI or concussion [14], our data do not permit causal inferences about the effectiveness of specific interventions within this cohort. To date, no conclusive studies have demonstrated a specific treatment with clearly proven efficacy for CVI [24]. Nevertheless, in the case of CVI, establishing the diagnosis is crucial to make appropriate environmental adjustments [13].

### 4.1. Limitations

Based on the retrospective nature of this study, not all information may have been available for analysis. Examinations were often performed without parents. Therefore, not all records provided information about the onset, duration and possible improvement of visual dysfunction. Preexisting examination at the primary hospital prior to admission to the inpatient rehabilitation was not always accessible. Thus, comparison of visual function and judgement about rehabilitation progress cannot be drawn from the data available. Of the 284 planned consultations, 55 were not included in the analysis, either because consent for study participation was not obtained or because parents were not contacted (10/55) in cases where the child had died. This introduces the possibility of selection bias. Children with more severe clinical courses, including those who died during or shortly after hospitalisation, may be underrepresented in the analysed cohort.

Age distribution was largely balanced across the cohort, except for the 18–20‐year age group, which was least represented (2/163), likely due to primary admission to adult rehabilitation services at this age. This may limit the applicability of the findings to older adolescents and young adults.

Distinction of acquired or preexisting condition was made based on the available data. Untreated amblyopia, refractive errors or latent strabismus may not have been known or diagnosed prior the hospitalisation in the inpatient rehabilitation. In some cases, a definitive ophthalmological diagnosis could not be made retrospectively based on the documentation. In others, only clinical findings were recorded without a conclusive note. In these patients, the diagnosis was made based on the available data. This presented specific challenges in diagnosing CVI, as it was not specifically investigated at the ophthalmic rehabilitation visit. Additional extended neuropsychological workup as part of the CVI diagnosis was not yet possible because of the general condition during the rehabilitation process. The absence of comprehensive neuropsychological assessment limits diagnostic certainty and may affect the interpretation and generalisability of the findings. Advanced visual function or morphological testing such as visual field evaluation or OCT, respectively, were not available to further support the evaluation and diagnosis. However, most children presented in an unstable general health condition at the time of the examination, precluding these specific tests at the time of consultation. Furthermore, children with visual dysfunction received a separate low vision assessment during rehabilitation. This assessment was not part of the ophthalmological evaluation by our team and was therefore excluded from the present analysis.

## 5. Conclusion

The findings of this study are primarily generalisable to other paediatric centres receiving referrals for children with a broad spectrum of underlying neurological and medical conditions. The results of this study indicate that a substantial proportion of children referred with suspected visual dysfunction are found to have clinically relevant ophthalmological abnormalities. These findings underline the importance of offering comprehensive ophthalmological assessment to all children with suspected visual dysfunction to enable targeted therapeutic interventions and effectively support the rehabilitation process. Further studies should be conducted in a prospective manner with a standardised protocol and assessment to allow more systematic evaluation of prevalence, diagnostic pathways and potential therapeutic outcomes within the paediatric inpatient rehabilitation setting.

## Author Contributions

Milena Geissbühler: data curation, formal analysis, methodology, and writing–original draft and review and editing. Brigitte Simonsz‐Toth: conceptualisation, formal analysis, validation, and writing–review and editing. Andreas Meyer‐Heim: conceptualisation, data curation, formal analysis, methodology, and writing–review and editing. Christina Gerth‐Kahlert: conceptualisation, data curation, formal analysis, methodology, project administration, supervision, validation, and writing–review and editing.

## Funding

No funding was received. Open‐access publishing was facilitated by Universitat Zurich, as part of the Wiley–Universitat Zurich agreement via the Consortium of Swiss Academic Libraries.

## Disclosure

This work was presented at the 50th annual meeting of the European Paediatric Ophthalmology Society, Leiden, 09 October 2025. A previous version of the abstract from this work was published in the EPOS 2025 Abstractbook.

All authors agree to be accountable for all aspects of the work.

## Conflicts of Interest

The authors declare no conflicts of interest.

## Data Availability

The data that support the findings of this study are available on request from the corresponding author. The data are not publicly available due to privacy or ethical restrictions.
